# Depression among women of reproductive age in rural Bangladesh is linked to food security, diets and nutrition

**DOI:** 10.1017/S1368980019003495

**Published:** 2020-03

**Authors:** Thalia M Sparling, Jillian L Waid, Amanda S Wendt, Sabine Gabrysch

**Affiliations:** 1 Epidemiology and Biostatistics Unit, Heidelberg Institute of Global Health, Heidelberg University, Im Neuenheimer Feld 324, 69120 Heidelberg, Germany; 2 Innovative Metrics and Methods for Agriculture and Nutrition Actions (IMMANA), Friedman School of Nutrition Science & Policy, Tufts University, Boston, MA, USA; 3 Helen Keller International, Dhaka, Bangladesh; 4 Potsdam Institute for Climate Impact Research, Potsdam, Germany; 5 Charité – Universitätsmedizin Berlin, Berlin, Germany

**Keywords:** Food, Nutrition, Dietary diversity, Maternal health, Depression, Peripartum depression

## Abstract

**Objective:**

To quantify the relationship between screening positive for depression and several indicators of the food and nutrition environment in Bangladesh.

**Design:**

We used cross-sectional data from the Food and Agricultural Approaches to Reducing Malnutrition (FAARM) trial in Bangladesh to examine the association of depression in non-peripartum (NPW) and peripartum women (PW) with food and nutrition security using multivariable logistic regression and dominance analysis.

**Setting:**

Rural north-eastern Bangladesh.

**Participants:**

Women of reproductive age.

**Results:**

Of 2599 women, 40 % were pregnant or up to 1 year postpartum, while 60 % were not peripartum. Overall, 20 % of women screened positive for major depression. In the dominance analysis, indicators of food and nutrition security were among the strongest explanatory factors of depression. Food insecurity (HFIAS) and poor household food consumption (FCS) were associated with more than double the odds of depression (HFIAS: NPW OR = 2·74 and PW OR = 3·22; FCS: NPW OR = 2·38 and PW OR = 2·44). Low dietary diversity (<5 food groups) was associated with approximately double the odds of depression in NPW (OR = 1·80) and PW (OR = 1·99). Consumption of dairy, eggs, fish, vitamin A-rich and vitamin C-rich foods was associated with reduced odds of depression. Anaemia was not associated with depression. Low BMI (<18·5 kg/m^2^) was also associated with depression (NPW: OR = 1·40).

**Conclusions:**

Depression among women in Bangladesh was associated with many aspects of food and nutrition security, also after controlling for socio-economic factors. Further investigation into the direction of causality and interventions to improve diets and reduce depression among women in low- and middle-income countries are urgently needed.

Women of reproductive age have a high risk of developing depression, and women of every age are twice as likely to experience depression as men^(1)^. Peripartum depression, also commonly referred to as maternal or perinatal depression, is defined as depression during pregnancy and up to 1 year postpartum. It is a common morbidity during pregnancy and lactation and can have severe and long-lasting consequences for women and their children, including poor care for oneself and others and health effects on children^(2–8)^.

In high-income countries, pooled point prevalences for both minor and major depressive disorders in the peripartum period range from 6·5 to 13 %^(9)^. For low- and middle-income countries (LMIC), Fisher *et al*. reported a pooled depression prevalence of 16 % during pregnancy from nine countries and almost 20 % in the first year after birth from seventeen countries^(10)^. In South Asia (Pakistan, Nepal, India and Bangladesh), there is no representative population prevalence estimate for depression in any of the countries. We identified four studies that reported prevalence of depression in their non-representative peripartum samples, ranging from 18 to 52 %^(11–14)^.

The aetiology of depression is complex and much of it is still not well understood. Many factors may contribute to depression, most notably a history of mental illness, poor social support, experience of instability and violence, and hormonal changes during pregnancy and childbirth^(10)^, and possibly socio-economic status and poor physical health^(15)^. The risk of depression is most likely exacerbated by social stresses that are more prevalent in LMIC^(16–19)^. In a prospective cohort from southern Bangladesh, Gausia *et al*. found that depression during the current pregnancy, past mental illness, domestic fighting, poor relationship with the mother-in-law and death of a child predicted depression at 6 to 8 weeks postpartum^(12)^.

Nutrients and diet have also been hypothesized to contribute to the onset of depressive symptoms, as certain nutrients play a critical role in neurotransmission and mood regulation^(20–22)^. Nutrition may be especially related to depression in the peripartum period when additional demands on a woman’s body contribute to nutrient deficiencies. An association between food insecurity and depression has been observed in many contexts^(23–28)^. These other aspects of food consumption and diets, such as food insecurity or dietary quality and diversity, may also contribute to depression both through their contributions to nutrient intake and through the stresses related to food acquisition.

Some evidence also exists for a link of depression with diet^(29)^ and with micronutrient status^(30)^; for example, diets high in fish, healthy or Mediterranean diets, higher levels of vitamin D and PUFA may be protective against depression. However, the existing body of evidence is limited by the paucity of studies in populations with nutritional deficiencies. Bangladesh suffers from a very high prevalence of food insecurity and malnutrition. In 2014, almost one-quarter of all households in the country were food insecure; over half of non-peripartum women consumed diets inadequate in macro- and micronutrients and about 40 % of adult women were chronically energy deficient^(31)^,defined as BMI < 18·5 kg/m^2^. In 2011, a national survey found widespread deficiencies in zinc, iodine, iron, vitamin B_12_ and folate^(32)^.

Previous research has identified a gap in rigorously conducted and sufficiently powered studies on nutrition and depression in LMIC^(10^^,^^29^^,^^30^^)^. Given the high prevalence of dietary deficiencies, as well as food insecurity and other concomitant stressors, Bangladesh is an ideal place to investigate these relationships. The aim of the present study was to examine the association of screening positive for depression with several indicators of the food and nutrition environment in Bangladesh, including food insecurity, household food consumption, dietary diversity, anaemia and BMI, in both non-peripartum and peripartum women. We present peripartum and non-peripartum women separately, both because cut-offs of key indicators differ and because there is no clear hypothesis based on existing literature of how these groups differ in this setting. We do not aim to compare these groups explicitly.

## Methods

### Setting

In Sylhet division in north-eastern Bangladesh, malnutrition prevalence is above the national average, with 50 % of children under 5 years of age stunted and 41 % of pregnant women undernourished. Less than one-quarter of young children in Sylhet receive an minimally adequate diet^(31)^. In the Haor region of Sylhet, a flooding zone, much of the arable land is under water during the rainy season. Wealth disparities, gender inequality and fertility are also higher in Sylhet than in other areas of Bangladesh^(33–35)^. In this region, joint households of several brothers and their wives living together with the brothers’ parents is not uncommon, as is migratory and seasonal labour that takes working-aged men away from the homestead for extended periods of time.

The Food and Agricultural Approaches to Reducing Malnutrition (FAARM) study (registered at clincicaltrials.gov, identifier NCT02505711) is a cluster-randomized controlled trial located in thirteen unions of Habiganj district in Sylhet division^(36)^. The trial aims to assess the impact of an enhanced homestead food production (eHFP) programme, implemented by the international non-governmental organization Helen Keller International, on stunting in children under 36 months of age. During the baseline survey in 2015, FAARM enrolled 2623 women and their children in ninety-six geographic clusters. Half the clusters were randomized to receive a 3-year intensive eHFP programme. The present study is a planned secondary analysis of the FAARM baseline survey.

### Participation

Women were eligible for both the trial and the current analysis if they were married, their husband stayed overnight at the household at least once per year, they reported to be less than 30 years of age,* they had access to at least 40 m^2^ of land that stayed dry at least some months of the year and they expressed interest in taking part in an eHFP programme. If multiple women living in the same household fit these criteria, all were invited to participate.

Following an extensive explanation of the study design and participation requirements, the field managers obtained written informed consent to participate in the study. We conducted three interviews as part of the baseline survey. The first questionnaire gathered information about the household structure, the second collected information specific to the woman herself, in private as much as possible, and the third took anthropometric measurements and collected capillary blood from the woman and her child. Of the 2623 women who enrolled in FAARM, 2599 women (99 %) completed at least the household, women’s and anthropometry sections of the baseline survey.

### Depression

The Edinburgh Postpartum Depression Scale (EPDS) is a ten-item psychometric screening tool used to identify probable minor and major depression. The recall period is the last 7 d and respondents are asked to rank the items generally as they are relevant to the last 7 d. The range of the scale is 1–30, with a score of 1 signifying the lowest and a score of 30 signifying the highest likelihood of depression^(37)^.

The EPDS has been validated in many contexts for depression screening, including for pregnant and postpartum women, as it excludes the somatic symptoms of pregnancy. The EPDS was translated into Bangla^(38)^ and validated against the Structured Clinical Interview for Diagnosis of Depression (SCID-DSM-IV)^(39)^. Several studies from South Asia use a score of ≥12, which corresponds more closely to a diagnosis of major depressive disorder and has a higher positive predictive value for depression than lower cut-offs^(37)^. We therefore used an EPDS cut-off of 12 to screen for major depression, and a cut-off of 10 for minor depression in a sensitivity analysis^(19)^. We also adjusted some terms in the Bangla questionnaire for greater clarity, in line with suggestions of local Bangladeshi researchers.

### Food and nutrition security indicators

We analysed five measures of food and nutrition security. The two household measures of food access were: (i) the Household Food Insecurity Access Scale (HFIAS) from the Food and Nutrition Technical Assistance Project^(40)^, collapsed into three categories (food secure; mild or moderately food insecure; severely food insecure) since only 10 % of our population was moderately food insecure; and (ii) the World Food Programme’s Food Consumption Score (FCS), adapted for the Bangladeshi dietary pattern^(41)^, categorized into acceptable or unacceptable (borderline and poor) household consumption, since 86 % of the study population fell into the two ‘acceptable’ categories. (iii) Women’s nutrient adequacy^(31)^ was measured using 24 h recall of food groups contained in the thirteen-food-group model validated in Bangladesh^(42)^, together with the dichotomous cut-off used in the thirteen-group Women’s Dietary Diversity Score (WDDS), including the 15 g restriction^(43)^. (iv) Anaemia was identified using capillary blood analysed on Hemocue® 201+ machines. Non-pregnant women, whether lactating or not, with Hb < 12·0 g/dl and pregnant women with Hb < 11·0 g/dl, unadjusted for trimester, were classified as anaemic. (v) Height and weight of women were captured on Seca mobile stadiometers and scales. Women with BMI < 18·5 kg/m^2^ were classified as chronically energy deficient. In line with Demographic and Health Survey guidelines, we did not analyse BMI for pregnant women or women within the first 2 months after birth^(44)^. For further description of the indicators of food and nutrition security, see Supplemental File S1 in the online supplementary material.

### Potential confounders

During the survey, interviewers ascertained age in years, age at first marriage, years of completed education and a detailed birth history as self-reported by the index woman. Literacy was determined by asking the index woman to read a simple sentence. We asked an adult representative of each household the religion of the household head, the size of the household, and if anyone in the household owned a set list of assets in order to construct a wealth index^(31)^. In the analysis, we adjusted for several known risk factors for depression, especially lack of women’s agency (decision making, social support, communication and mobility), recent stillbirth, poverty, illiteracy and lack of education^(45)^. Factors such as intimate partner violence, having a history of depression or other difficulties in pregnancy and birth were either unavailable from the baseline data or were not applicable to all women (e.g. those never having given birth), and were therefore not included in the analysis. Other potential confounders were also included, such as age, age at first marriage, religion, household size, live births and interviewer. Potential confounders were categorized into evenly distributed or logical groups. The definition of all variables included is provided in Supplemental File S1 and Supplemental Table S1 in the online supplementary material.

### Statistical analysis

The FAARM trial was powered in relation to the primary outcome, length-for-age in children under 3 years old, accounting for intracluster correlation and loss to follow-up. As depression is relatively common, as are most of the food access and nutritional status indicators, the FAARM cross-sectional baseline sample provides high power to detect associations between these variables in an observational analysis. The sample was divided into peripartum and non-peripartum women and still yielded sufficient precision to make conclusions about the strength of associations observed. In the non-peripartum women’s sample, a *post hoc* power calculation based on the given sample size (*n* 1559) had over 95 % power for detecting simple correlations greater than 0·1. Generally, based on *z* tests for multiple logistic regression, with a sample size of 1000 there is 95 % power to detect an OR of 1·3.

We defined women as pregnant if they self-identified as pregnant during the interview or during the anthropometry or blood sampling (which was often slightly later). Postpartum status was defined as having given birth within the past 12 months. The distributions of depression, nutrition measures and other covariates were very similar between pregnant and postpartum women, and these were thus combined into peripartum women. All other women were classified as non-peripartum.

The few women with missing values were omitted. The number of missing values varied for each independent variable. Ninety-five women (4 %) of the total sample were either unavailable or refused blood collection at baseline, which resulted in missing values for anaemia. In the final adjusted analysis, there were two missing values from dietary diversity scales, three missing for time since most recent birth and nine missing BMI measurements. There was no evidence that women with missing values were systematically different from the remaining sample in terms of socio-economic measures and risk of depression.

Some variables were decided *a priori* to be included as covariates in multivariable models (age, religion, wealth, total live births, stillbirths in the last year, breast-feeding and recent births). Other potential confounders were tabulated against the food and nutrition security measures and against the depression outcome (in the full sample) and we used Pearson *χ*^2^ tests to see whether they were associated with depression at a significance level of *P* < 0·1. Crude associations of each nutritional measure with depression were estimated in multilevel random-effects logistic regression models (base models), accounting for clustering at the village level. Potential confounders (*a priori* and those associated with depression: marital age, years since first marriage, number of household members, woman’s education, woman’s literacy, woman’s social support, decision making, mobility and communication, and interviewer) were then added to the base model (Model 1). Finally, we added the other food and nutrition indicators as potential confounders (apart from anaemia since this would have dropped the ninety-three women with missing values and there was no crude association with depression) to each of the models (Model 2). For instance, in the regression model of women’s dietary diversity on depression, we added food insecurity, household food consumption and BMI for non-peripartum women.

Lastly, we conducted a dominance analysis, which provides a ranking of explanatory power of all exposure variables in terms of depression. For this, we fitted logistic regression models including all food and nutrition security variables and confounders (treated as categorical variables) and used pseudo *R*^2^ statistics to assess goodness-of-fit^(46)^. For several regression models including the dominance analyses, small clusters with few observations per interviewer were grouped with the nearest cluster, and interviewers who completed few questionnaires were grouped with their partner interviewer to avoid missing data due to homogeneous outcomes within groups. The statistical software package Stata version 14.0 or 15.0 was used for all analyses.

## Results

**Fig. 1:**
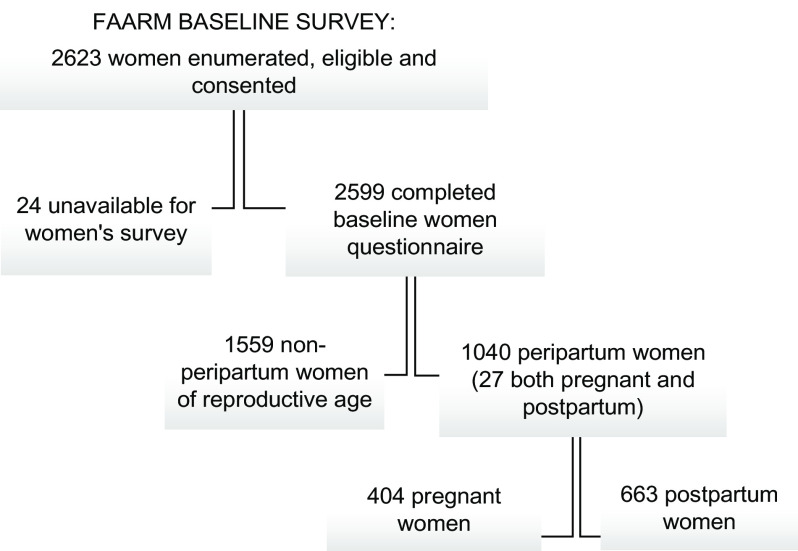
Flowchart of the study sample women of reproductive age participating in the Food and Agricultural Approaches to Reducing Malnutrition (FAARM) trial

Of the 2599 women included in analysis, 1040 (40 %) were peripartum (pregnant or postpartum; Fig. 1). Table 1 presents the prevalence of depression and various indicators of food and nutrition security, separately for non-peripartum and peripartum women. Twenty per cent of women (*n* 527) screened positive for major depression (EPDS score of ≥12) and 31 % (*n* 802) screened positive for at least minor depression (EPDS score of ≥10). Screening positive for depression was slightly more prevalent in non-peripartum women (22 %) than in peripartum women (18 %).

**Table 1:** Prevalence of depression and indicators of food and nutrition security among women of reproductive age (*n* 2599) participating in the Food and Agricultural Approaches to Reducing Malnutrition (FAARM) trial in rural north-eastern Bangladesh, 2015

	Non-peripartum women (*n* 1559)		Peripartum women (*n* 1040)	
	*n*	%	*n*	%
Depression				
Major depression (EPDS ≥ 12)	338	22	189	18
Minor or major depression (EPDS≥10)	510	33	292	28
Food and nutrition security				
Household indicators				
Food insecurity (HFIAS)				
Food secure	374	24	251	24
Mildly/moderately food insecure	557	36	387	37
Severely food insecure	628	40	402	39
Poor household food consumption (FCS < 43/112)	217	14	134	13
Women’s indicators				
Inadequate dietary diversity (WDDS < 5/13 groups)*	966	62	669	64
Food groups (WDDS 13 groups, previous 24 h)				
Dairy	437	28	309	30
Eggs	210	13	129	12
Fish	1225	79	841	81
Meat	138	9	97	9
Dark-green leafy vegetables	430	28	252	24
Vitamin A-rich fruit and/or vegetables	299	19	179	17
Vitamin C-rich fruit and/or vegetables	832	53	537	52
Women’s anthropometry and anaemia				
CED (BMI < 18·5 kg/m^2^)*	535	35	200†	37
Women’s BMI*				
Severely or moderately thin (BMI < 17·0 kg/m^2^)	229	15	82	13
Mildly thin (BMI = 17·0–18·49 kg/m^2^)	306	20	133	21
Normal (BMI = 18·5–24·99 kg/m^2^)	763	49	330	53
Overweight and obese (BMI ≥ 25·0 kg/m^2^)	252	16	76	12
Anaemia (non-pregnant, Hb < 12 g/dl; pregnant, Hb <11 g/dl)*	441	29	405	41

EPDS, Edinburgh Postpartum Depression Scale; HFIAS, Household Food Insecurity Access Scale; FCS, Food Consumption Score; WDDS, Women’s Dietary Diversity Score, thirteen-group index with 15 g cut-off; CED, chronic energy deficiency.

* Missing values: WDDS, *n* 2; CED, *n* 9; BMI, *n* 9; anaemia in non-peripartum women, *n* 54; anaemia in peripartum women, *n* 41.

† BMI not applicable for pregnant women or women less than 2 months postpartum: women 2–12 months postpartum, *n* 593.

Forty per cent of women and their respective households were classified as severely food insecure during the previous month and another 36 % of women were mildly to moderately food insecure. Fourteen per cent of households had food consumption scores indicating poor diets in the previous 7 d. Nearly two-thirds of women (63 %) consumed fewer than five food groups out of thirteen on the previous day, signifying a less than 50 % probability of meeting micronutrient needs in the diet. The linear dietary diversity score (using thirteen food groups) was approximately normally distributed around a mean of 4·1 (sd 1·5). Hb levels were also normally distributed, with a mean Hb concentration of 12·3 (sd 1·3) g/dl. Twenty-nine per cent of non-peripartum women were anaemic (Hb < 12 g/dl), while 39 % of pregnant women (Hb < 11 g/dl) and 42 % of postpartum women (Hb < 12 g/dl) were anaemic. Among non-peripartum women, 35 % were mildly to severely underweight (BMI < 18·5 kg/m^2^), and thus considered chronically energy deficient, and 15 % had BMI < 17·0 kg/m^2^. The mean BMI (excluding pregnant women and those 2 months postpartum) was 20·0 (sd 3·1) kg/m^2^.

Demographic and socioeconomic characteristics were similar in non-peripartum and peripartum women, and thus are presented for the whole population in Table 2. Sixty-four per cent of women were married prior to their nineteenth birthday; 26 % were married by age 16 years or younger. Mean current age was 25 years. Women were often much younger than their husbands, with almost 70 % being ≥6 years younger and 25 % being >10 years younger. Seven per cent of households had more than one eligible woman.

**Table 2:** Descriptive characteristics of the study population of women of reproductive age (*n* 2599) participating in the Food and Agricultural Approaches to Reducing Malnutrition (FAARM) trial in rural north-eastern Bangladesh, 2015

Variable	*n*	%	Variable	*n*	%
Woman’s age (years)			Woman’s education		
15–18	162	6	None	440	17
19–21	503	20	Partial primary	558	21
22–25	865	33	Primary	613	24
26–30	858	33	Partial secondary	841	32
≥31	211	8	Secondary or more	147	6
Age at first marriage (years)			Live births		
≤16	678	26	0	301	12
17–18	990	38	1	773	30
19–20	586	23	2	749	29
≥21	345	13	≥3	776	30
Years since first marriage			Stillbirths in last year		
≤2	481	19	No	2565	99
3–4	467	18	Yes	34	1
5–6	444	17			
7–8	402	15	Currently breast-feeding	1418	55
9–10	343	13			
>10	462	17	Social support		
Religion			Little (0–3 points)	433	17
Muslim	1787	69	Some (4 points)	615	24
Hindu	812	31	A lot (5 points)	1551	60
Wealth			Decision-making ability		
Quintile 1 (poorest)	654	25	Unable (0 points)	661	25
Quintile 2	565	22	Little (1 point)	646	25
Quintile 3	508	20	Some (2 points)	566	22
Quintile 4	460	18	High (3–6 points)	726	28
Quintile 5 (richest)	412	16	Mobility outside home		
Household size			Unable (0 points)	572	22
1–4 members	644	25	May be able (1 point)	1027	40
5–9 members	1395	54	Somewhat able (2 points)	548	21
≥10 members	560	22	Able (3 points)	452	17
Woman’s literacy			Communication		
Literate	1683	65	Unable or little (0–4 points)	631	24
Partially literate	244	9	Somewhat able (5 points)	1214	46
Illiterate	672	26	Able (6–8 points)	754	29

Social support seemed the strongest form of agency for most women, with 60 % having a lot of social support. Roughly a quarter of women fell into each of the four levels of decision making, with 25 % of women unable to decide on any household matters. In the mobility domain, 22 % of women were unable to travel alone or with children outside the homestead at all, and only 17 % had the ability to freely travel away from the homestead. Twenty-four per cent of women communicated either never or very rarely with their husband or with other women about issues. The detailed results of the agency scores are shown in the online supplementary material, Supplemental Table S2.

**Fig. 2:**
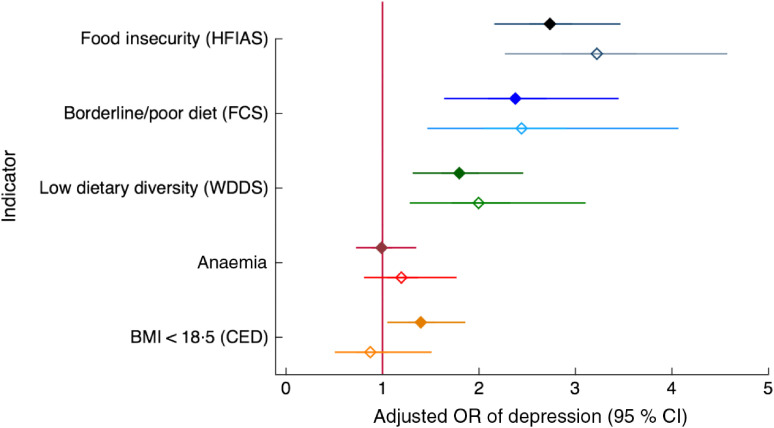
Relationship of food and nutrition security indicators with depression among women of reproductive age (*n* 2599) participating in the Food and Agricultural Approaches to Reducing Malnutrition (FAARM) trial in rural north-eastern Bangladesh, 2015. Odds ratios of depression, with 95 % confidence intervals represented by horizontal bars, with indicators of food and nutrition security are shown for non-peripartum women (NPW; 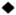) and peripartum women (PW; 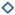), adjusted for age, age at first marriage, time since first marriage, religion, wealth, household size, live births, woman’s education, literacy, breast-feeding status, woman’s agency in four domains (mobility, support, decision making and interpersonal communication), birth within last 3 years, time since most recent birth and data collection officer (Model 1). HFIAS, Household Food Insecurity and Access Scale (per one-category increase; NPW, *n* 1568; PW, *n* 997); FCS, Food Consumption Score (NPW, *n* 1568; PW, *n* 997); WDDS, Women’s Dietary Diversity Score (NPW, *n* 1566; PW, *n* 997); Anaemia (NPW, *n* 1513; PW, *n* 961); BMI, BMI in kg/m^2^ (NPW, *n* 1559; PW, *n* 520*); CED, chronic energy deficiency. *BMI for PW includes only women between 2 and 12 months postpartum

All measures of food and nutrition security except anaemia were associated with depression in the crude model and they generally remained significantly associated with depression also when adjusting for a range of potential confounders (Model 1), as well as for other measures of food and nutrition security (Model 2; Fig. 2 and online supplementary material, Supplemental Table S3). In the dominance analysis, after geographic cluster and interviewer, household food insecurity and household food consumption were the strongest explanatory variables for depression, followed by social support and low dietary diversity, then age, mobility, age at first marriage and wealth quintile, in that order. In the peripartum model, results were similar with food insecurity as the strongest explanatory variable after cluster and interviewer, followed by social support, household food consumption, low dietary diversity of women, religion, age at first marriage, education and wealth (Table 3).

**Table 3:** Dominance analysis results: explanatory power of each factor to screening positive for depression among women of reproductive age (*n* 2599) participating in the Food and Agricultural Approaches to Reducing Malnutrition (FAARM) trial in rural north-eastern Bangladesh, 2015

Contributing variable	Non-peripartum women (*n* 1440)* Overall fit statistic (pseudo *R*^2^) = 0·2654		Peripartum women (*n* 1040)* Overall fit statistic (pseudo *R*^2^) = 0·2898	
	Rank	Relative contribution to overall fit	Rank	Relative contribution to overall fit
Cluster	1	0·0747	1	0·0797
Interviewer	2	0·0708	2	0·0748
Household food insecurity (HFIAS)	3	0·0480	3	0·0599
Household food consumption (FCS)	4	0·0165	5	0·0110
Social support	5	0·0087	4	0·0146
Low dietary diversity (<5/13 groups)	6	0·0070	6	0·0099
Age	7	0·0070	13	0·0042
Mobility	8	0·0066	11	0·0046
Age at first marriage	9	0·0065	8	0·0063
Wealth quintile	10	0·0043	10	0·0048
CED (BMI < 18·5 kg/m^2^)	11	0·0031	not used for peripartum women	
Live births	12	0·0031	12	0·0045
Interpersonal communication	13	0·0023	15	0·0007
Religion	14	0·0023	7	0·0065
Education	15	0·0021	9	0·0050
Household size	16	0·0015	14	0·0027
Decision-making capacity	17	0·0009	16	0·0006
Anaemia	18	0·0001	not used for peripartum women	

HFIAS, Household Food Insecurity and Access Scale; FCS, Food Consumption Score; CED, chronic energy deficiency.

* Missing values. NPW: ninety-three observations dropped due to missing values for anaemia; two observations dropped due to missing dietary diversity values; nine observations dropped due to missing BMI. PW: three observations dropped due to missing time since birth information.

All potential confounders were crudely associated with depression in the non-peripartum sample or the peripartum sample or both, but very few factors stayed associated with depression in the adjusted models. More social support, higher wealth and younger age, as well as older age at first marriage and less mobility (ability to travel outside the homestead alone), were related to lower odds of depression in many adjusted models.

**Fig. 3:**
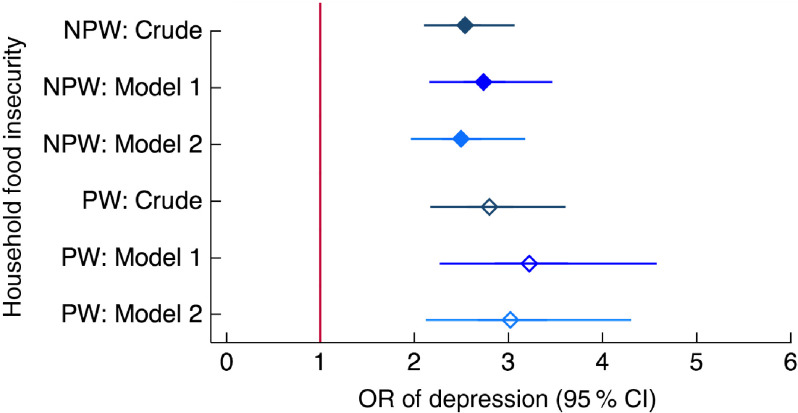
Relationship between household food insecurity and depression among women of reproductive age (*n* 2599) participating in the Food and Agricultural Approaches to Reducing Malnutrition (FAARM) trial in rural north-eastern Bangladesh, 2015. Odds ratios of depression, with 95 % confidence intervals represented by horizontal bars, from (crude and adjusted) multilevel models with three levels of household food insecurity (assessed using the Household Food Insecurity Access Scale (HFIAS)) in non-peripartum women (NPW; 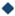) and peripartum women (PW; 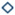). Crude model (NPW, *n* 1559; PW, *n* 1040); Model 1 (NPW, *n* 1559; PW, *n* 1037) is adjusted for age, age at first marriage, time since first marriage, religion, wealth, household size, live births, stillbirths in last year, woman’s education, literacy, breast-feeding status, woman’s agency in four domains (mobility, support, decision making and interpersonal communication), birth within last 3 years, time since most recent birth and data collection officer; Model 2 (NPW, *n* 1548; PW, *n* 1037) is additionally adjusted for household food consumption (assessed using the Food Consumption Score (FCS)), women’s dietary diversity (assessed using the thirteen-group Women’s Dietary Diversity Score (WDDS): <5/13 groups *v*. ≥5/13 groups) and BMI for NPW

**Fig. 4:**
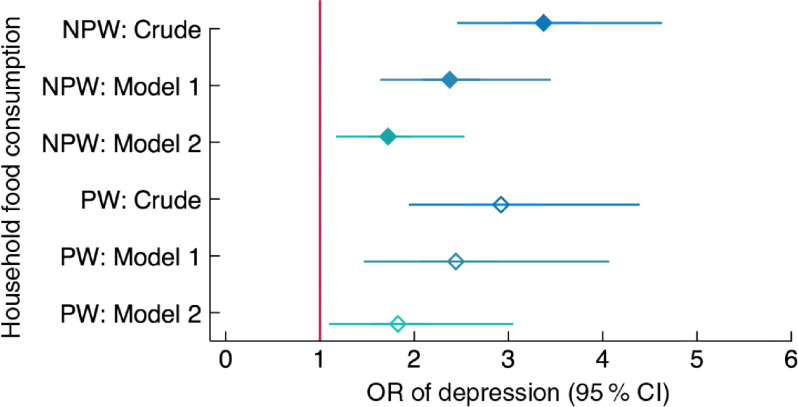
Relationship between household food consumption and depression among women of reproductive age (*n* 2599) participating in the Food and Agricultural Approaches to Reducing Malnutrition (FAARM) trial in rural north-eastern Bangladesh, 2015. Odds ratios of depression, with 95 % confidence intervals represented by horizontal bars, from (crude and adjusted) multilevel models with household food consumption (assessed using the Food Consumption Score (FCS)) in non-peripartum women (NPW; 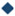) and peripartum women (PW; 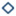). Crude model (NPW, *n* 1559; PW, *n* 1040); Model 1 (NPW, *n* 1559; PW, *n* 1037) is adjusted for age, age at first marriage, time since first marriage, religion, wealth, household size, live births, stillbirths in last year, woman’s education, literacy, breast-feeding status, woman’s agency in four domains (mobility, support, decision making and interpersonal communication), birth within last 3 years, time since most recent birth and data collection officer; Model 2 (NPW, *n* 1548; PW, *n* 1037) is additionally adjusted for household food insecurity and access (assessed using the Household Food Insecurity Access Scale (HFIAS)), women’s dietary diversity (assessed using the thirteen-group Women’s Dietary Diversity Score (WDDS): <5/13 groups *v*. ≥5/13 groups) and BMI for NPW

Of all the food and nutrition security indicators, household-level food insecurity (HFIAS) and household food consumption score (FCS) were most strongly related to depression, both in the adjusted regression models and in the dominance analysis. Each additional level of food insecurity (from none to mild/moderate and from mild/moderate to severe) increased the odds of depression more than twice in non-peripartum women, and more than three times in peripartum women (Fig. 3). Women living in households classified as having poor or borderline food consumption *v*. adequate food consumption had more than twice the odds of depression, even when accounting for food insecurity, anaemia status and BMI (Fig. 4).

**Fig. 5:**
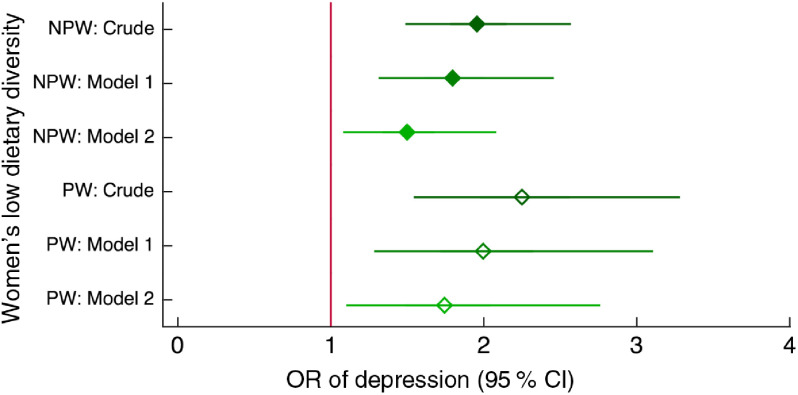
Relationship between low dietary diversity and depression among women of reproductive age (*n* 2599) participating in the Food and Agricultural Approaches to Reducing Malnutrition (FAARM) trial in rural north-eastern Bangladesh, 2015. Odds ratios of depression, with 95 % confidence intervals represented by horizontal bars, from (crude and adjusted) multilevel models with women’s dietary diversity (assessed using the thirteen-group Women’s Dietary Diversity Score (WDDS): <5/13 groups *v*. ≥5/13 groups) in non-peripartum women (NPW; 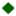) and peripartum women (PW; 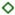). Crude model (NPW, *n* 1557; PW, *n* 1040); Model 1 (NPW, *n* 1557; PW, *n* 1037) is adjusted for age, age at first marriage, time since first marriage, religion, wealth, household size, live births, stillbirths in last year, woman’s education, literacy, breast-feeding status, woman’s agency in four domains (mobility, support, decision making and interpersonal communication), birth within last 3 years, time since most recent birth and data collection officer; Model 2 (NPW, *n* 1548; PW, *n* 1037) is additionally adjusted for household food insecurity and access (assessed using the Household Food Insecurity Access Scale (HFIAS)), household food consumption (assessed using the Food Consumption Score (FCS)) and BMI for NPW

Inadequate dietary diversity (fewer than five food groups out of thirteen) was associated with about twice the odds of depression compared with eating five or more food groups in the previous day (Fig. 5), and was ranked sixth in explaining depression among both non-peripartum and peripartum women.

**Fig. 6:**
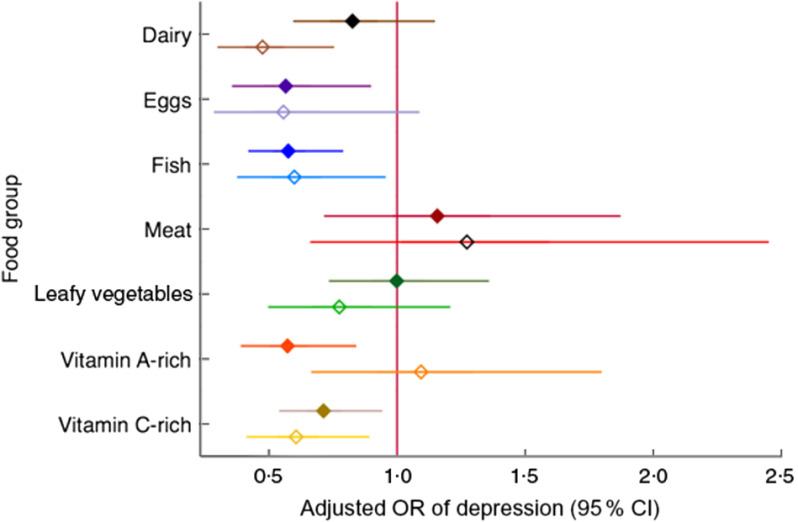
Relationship of depression with consuming specific nutrient-dense food groups among women of reproductive age (*n* 2599) participating in the Food and Agricultural Approaches to Reducing Malnutrition (FAARM) trial in rural north-eastern Bangladesh, 2015. Adjusted odds ratios, with 95 % confidence intervals represented by horizontal bars, in non-peripartum women (NPW; 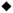) and peripartum women (PW; 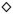), of screening positive for depression and consuming more than 15 g of certain food groups in the previous 24 h *v*. not consuming those food groups or consuming less than 15 g (Women’s Dietary Diversity Scale, thirteen groups): dairy, eggs, fish, flesh foods (‘meat’), dark-green leafy vegetables (‘leafy vegetables’), vitamin A-rich fruits and vegetables (‘vitamin A-rich’), vitamin C-rich fruits and vegetables (‘vitamin C-rich’). Each group is adjusted for age, age at first marriage, time since first marriage, religion, wealth, household size, live births, stillbirths in last year, woman’s education, literacy, breast-feeding status, woman’s agency in four domains (mobility, support, decision making and interpersonal communication), birth within last 3 years, time since most recent birth and data collection officer. Estimates to the left of 1 (null value) indicate a ‘protective’ association

Several individual food groups eaten in the previous day, namely eggs, fish, vitamin A-rich foods and vitamin C-rich foods, were associated with reduced odds of depression in non-peripartum women. Among peripartum women, eating dairy, eggs (borderline), fish and vitamin C-rich foods on the previous day was associated with lower odds of depression. Meat and dark-green leafy vegetables were the only food groups that were not associated with depression (Fig. 6 and online supplementary material, Supplemental Table S4).

**Fig. 7:**
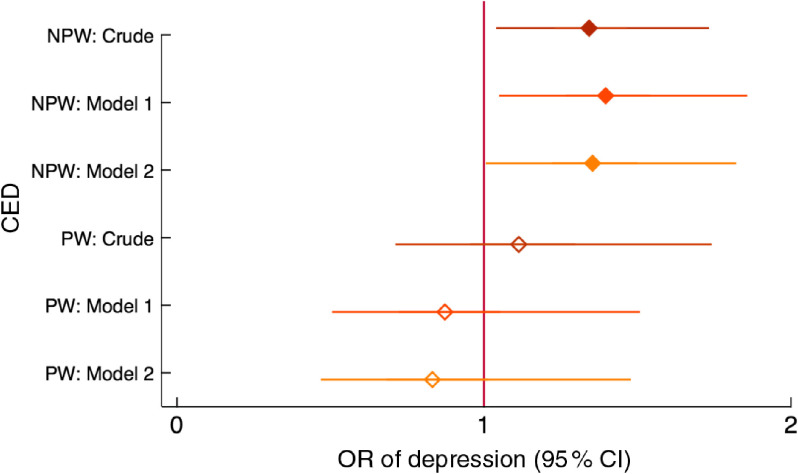
Relationship between chronic energy deficiency (CED) and depression among women of reproductive age (*n* 2599) participating in the Food and Agricultural Approaches to Reducing Malnutrition (FAARM) trial in rural north-eastern Bangladesh, 2015. Odds ratios of depression,with 95 % confidence intervals represented by horizontal bars, from (crude and adjusted) multilevel models with CED (BMI < 18·5 kg/m^2^) in non-peripartum women (NPW; 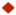) and peripartum women (PW; 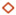). Crude model (NPW, *n* 1550; PW, *n* 550; only postpartum women between 2 and 12 months were included in PW); Model 1 (NPW, *n* 1550; PW, *n* 550) is adjusted for age, age at first marriage, time since first marriage, religion, wealth, household size, live births, stillbirth in last year, woman’s education, literacy, breast-feeding status, woman’s agency in four domains (mobility, support, decision making and interpersonal communication), birth within last 3 years, time since most recent birth and data collection officer; Model 2 (NPW, *n* 1548; PW, *n* 550) is additionally adjusted for household food insecurity and access (assessed using the Household Food Insecurity Access Scale (HFIAS)), household food consumption (assessed using the Food Consumption Score (FCS)) and women’s dietary diversity (assessed using the thirteen-group Women’s Dietary Diversity Score (WDDS): <5/13 groups *v*. ≥5/13 groups)

**Fig. 8:**
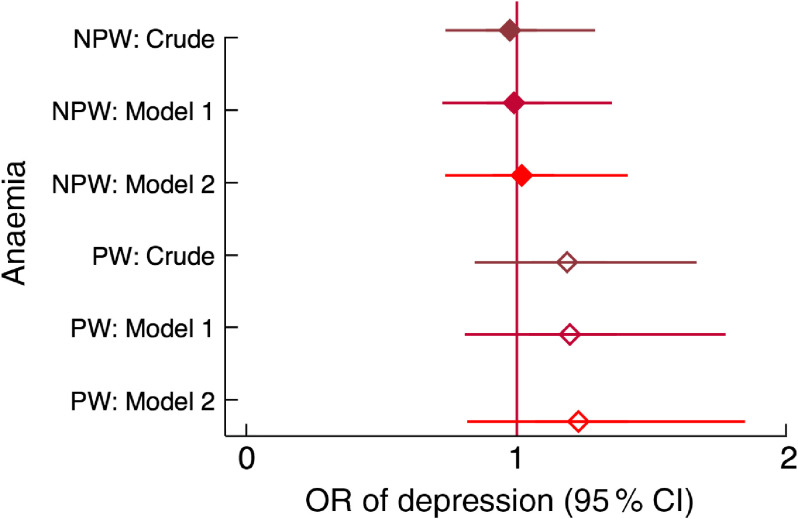
Relationship between anaemia and depression among women of reproductive age (*n* 2599) participating in the Food and Agricultural Approaches to Reducing Malnutrition (FAARM) trial in rural north-eastern Bangladesh, 2015. Odds ratios of depression, with 95 % confidence intervals represented by horizontal bars, from (crude and adjusted) multilevel models with anaemia in non-peripartum women (NPW; 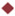) and peripartum women (PW; 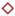). Crude model (NPW, *n* 1505; PW, *n* 999); Model 1 (NPW, *n* 1505; PW, *n* 999) is adjusted for age, age at first marriage, time since first marriage, religion, wealth, household size, live births, stillbirth in last year, woman’s education, literacy, breast-feeding status, woman’s agency in four domains (mobility, support, decision making and interpersonal communication), birth within last 3 years, time since most recent birth and data collection officer; Model 2 (NPW, *n* 1503; PW, *n* 961) is additionally adjusted for household food insecurity and access (assessed using the Household Food Insecurity Access Scale (HFIAS)), household food consumption (assessed using the Food Consumption Score (FCS)), women’s dietary diversity (assessed using the thirteen-group Women’s Dietary Diversity Score (WDDS): <5/13 groups *v*. ≥5/13 groups) and BMI for NPW

Chronic energy deficiency in non-peripartum women (BMI < 18·5 kg/m^2^) was associated with one-third higher odds of having depressive symptoms, and the effect size was not affected by adjustment for confounders. Adjusting for food and nutritional status variables (Model 2) widened the confidence interval and the association became borderline significant (Fig. 7). Anaemia was not associated with depression in any model (Fig. 8).

## Discussion

In this large, cross-sectional study from rural Bangladesh, we found that 20 % of women screened positive for major depressive disorder, which is high but comparable to other estimates in the region^(10^^,^^16^^,^^19^^,^^47^^)^. We further found that household food insecurity, poor household food consumption scores, women’s dietary diversity and to some degree low BMI were positively associated with depression in both peripartum and non-peripartum young women.

Certain food groups were associated with lower odds of depression, especially eggs, fish and vitamin C-rich foods. Eating vitamin A-rich foods showed almost no association with depression among peripartum women, whereas in non-peripartum women, vitamin A-rich food consumption was associated with about 40 % lower odds of depression. This may be due to the fact that vitamin A supplementation is in the routine package of interventions for lactating women in Bangladesh and thus vitamin A status may already be better during this time. There are very few studies from LMIC examining the relationship of specific vitamins, classes of nutrients or indices such as dietary diversity with depression. Two systematic reviews on the topic, based mainly on studies from high- and middle-income settings, suggested that certain nutrients and diets may be protective against depression, such as PUFA and high intake of fish^(29^^,^^30^^)^. In a meta-analysis, lower zinc and iron concentrations were both associated with depression^(48)^. As oxidative stress is linked to neurological disease, it is biologically plausible that consuming greater amounts of vitamin A, vitamin C, PUFA, fish or eggs could protect mental health^(49)^. Furthermore, from a sociocultural perspective, eating a more varied diet of nutritious and favourite foods (such as fish, vegetables and fruit) may also contribute to a greater sense of well-being.

Anaemia was not associated with depression in the present study, although it has been shown to be associated with postpartum depression elsewhere^(50)^. Anaemia can cause physical strain on the body, fatigue and various health issues, as well as being a proxy for micronutrient deficiencies, thus potentially predisposing to depression. However, the aetiology of anaemia in the Bangladeshi population is not straightforward and not necessarily due to iron deficiency^(51,52)^. Thalassaemia and other haemoglobinopathies may be a causal factor, which could mean that women are anaemic despite an adequate diet^(53^^,^^54^^)^. Furthermore, the high prevalence of chronic anaemia in our study population may mean that women have adjusted to low levels of Hb as a normal state and it may thus not be a differentiating factor for depression in this context.

The association between low BMI and screening positive for depression was not as strong as several other measures of food and nutrition security. While it is conceivable that food insecurity, poor food access and low dietary diversity would lead to low BMI in women, and these factors could also be independently associated with depression (via micronutrients leading to neurological changes), these factors did not confound the relationship, suggesting that they are independently related to depression. Thinness in Bangladesh may be a very weak proxy for women’s supply of micronutrients implicated in neurotransmission. In the case that causality runs the other direction (i.e. poor food and nutrition security causing depression), the weak link to BMI may be due to depression impacting dietary diversity and quality more than it impacts macronutrient intake or due to general fatigue that contributes to depression. In high-income settings, overweight is inconsistently linked to poor mental health^(55^^,^^56^^)^, but few studies exist on underweight or in the context of low-income countries. In non-urban, less globalized communities, thinness often carries a negative connotation, whereas fatness is a sign of health and happiness^(57)^. Given these different cultural constructions of body image, research findings from North America and Europe, even when they focus on low-income groups, are not comparable to Bangladesh.

In South Asia, women often have limited ability to make decisions, govern resources, engage in certain social activities or travel outside the homestead independently^(58)^. They are often expected to conform to certain norms as a wife and a mother, roles which are often subservient to their husband, father or mother-in-law. This impacts their ability to make choices about their daily activities, land use or cultivation, their food purchases, their own and their children’s health and diets, and their self-care and social support^(59–61)^. These dynamics can impact both the nutritional status of women and their family (e.g. serving the best and biggest portions of nutrient-rich foods to adult men), as well as women’s mental health and sense of agency (e.g. through feeling powerless or unable to make important health decisions)^(62)^. Women’s empowerment is therefore an important factor to consider in an analysis of nutrition and depression.

### Strengths and limitations

The present study has several advantages. The data come from a large sample, in which enrolment was clearly defined and participation was high. Although FAARM excluded households without enough land for gardening, the general characteristics of these young, married women living in a poor, rural setting most likely make the present study’s findings more generalizable than findings from clinical settings in previous studies. The assessment was carried out by well-trained and experienced data collectors, and completed within 3 months, therefore limiting heterogeneity that could have been introduced by longer assessment periods. We were able to examine many different aspects of the food environment and related health outcomes. We were also able to account for a wide and novel range of potential confounders of the relationships between indicators of food and nutrition security and depression, including dimensions of gender and women’s agency.

The most important limitation of the present study is the cross-sectional character of the data set, which means that we cannot rule out reverse causality. Based on previous evidence, we wanted to explore the effect of food and nutrition security on depression since some studies in high-income populations show that certain nutrients, such as PUFA or B vitamins, may be associated with better mental health^(30)^. Furthermore, healthier diets and food security have also been linked to better mental health in some settings^(29)^. These effects may be both physiological (e.g. through neurotransmitters) and/or through psychological and cultural pathways. However, given the cyclical and complicated nature of depressive symptomatology, it is entirely possible that depression causes poor self-care, poor care of others and contributes in other ways, such as through loss of motivation and ability to work, to worsening food and nutrition security. Although it is not possible to determine the causal direction of the relationships observed in the present study, the direction and magnitude of associations observed provide novel, robust evidence on which to base future studies. Longitudinal analyses that measure both nutrition and depression over time are needed so that baseline levels can be controlled for and onset can be determined.

The gold standard for diagnosing clinical depression is a lengthy interview conducted by a trained psychologist^(7)^and therefore screening tools are often preferred in population studies^(63^^,^^64^^)^. Our confidence in the results of the present and other studies rests on the assumption that the validation against a gold standard was thorough enough for the screening result to reasonably correspond to a depression diagnosis. The Bangladesh validation study suffered from several methodological issues, however. Very few participants (nine of 100) were diagnosed with (minor or major) depression, only three of which with major depression^(39)^, precluding the possibility of establishing a cut-off for major depression. Furthermore, the very low percentage of women diagnosed with any form of depression in the validation study calls into question either the very high prevalence of depression often detected using the EPDS or the validation methods used. Lastly, the validation study used a convenience sample drawn from an urban immunization clinic, which may not be generalizable to the rest of Bangladesh.

Shrestha *et al*. reviewed all EPDS validation studies in LMIC and found the methodological quality to be low overall, with the Bangladeshi validation study getting 6 out of 9 possible points^(65)^. Screening tools are validated against a clinical interview and although this is the current ‘gold standard’, clinical interview methods from high-income settings introduced in LMIC contexts may in fact not be appropriate or reliable unto themselves. Despite all these issues, any over- or under-ascertainment of depression introduced by the screening tool is likely to affect both women with good and bad nutrition to the same degree, and should thus not bias the odds ratios.

The present study is a secondary analysis in which we make multiple comparisons between a range of indicators of food and nutrition security and depression. Food frequency and dietary diversity modules can give a broad overview of diet quality and can capture complex synergies of ingested foods. However, these measures are self-reported and known to be imprecise and suffer from reporting bias^(66)^. In the case of the WDDS, the measure is not validated for individual dietary assessment of adequacy, but full dietary assessment through a lengthy quantitative food consumption questionnaire is not feasible in many research projects, and even these are not validated for individual assessment if they cover less than 30 d. Therefore, the WDDS is still the most widely used proxy for dietary quality. There is an ongoing debate about how to use the WDDS in general and how to adapt it so that it properly reflects the environment in which it is used^(67^^,^^68^^)^. Some researchers argue that the WDDS can be used as a proxy for food insecurity^(68)^. In the current analysis, these measures were correlated but not collinear, suggesting that when controlling for individual and household dietary diversity, the HFIAS captures stress and other non-diversity effects of food insecurity, and when controlling for all three, the long-term effects of low BMI and diet-related anaemia on depression are taken into account. It would be desirable to also use biological measures of micronutrient deficiency in future analyses. As we chose these various indicators as proxies of nutritional status, considering their different merits based on previous literature and conceptual frameworks, we felt that it was important to present each in association with depression, although this amounted to a large number of comparisons.

We tried to be comprehensive in our inclusion of potential confounders, but the potential for residual confounding is a limitation, as in any observational study. Although we account for some aspects of women’s empowerment in the current analysis and there remains a strong relationship between food and nutrition security and depression in adjusted analyses, other unmeasured aspects of women’s empowerment may still have an unmeasured influence on both mental health and food and nutrition security. Similarly to women’s empowerment, poverty could be associated with food insecurity and insufficient diets, as well as with poor mental health, and thus confound the relationship^(6)^. We used an asset index as a measure for poverty/wealth, which is more reliable than self-reported income in subsistence settings, but still only a proxy. Therefore, there may be residual confounding by poverty, exaggerating any effects on mental health by nutrition and food security.

### Practical implications

Depression prevalence in most countries is rising. It may be that this is due to more and better measurement and higher detection across a range of settings. Even if this is the case, a previously unaddressed and under-studied mental health burden has been revealed^(15)^. While the causal factors that lead to depression are still not well understood, any efforts to identify these causes, address them and reduce the burden of depression are important. Specifically, the social and economic determinants of mental health, including aspects of gender and poverty, warrant further study and increased policy and programmatic attention.

We provide some evidence that food and nutrition security are linked with depression, which may imply that improving food access, diets and nutritional status could contribute to improving mental health. At the same time, it could imply that depression is a cause of poor diets and nutrition in women, and also their children. In line with this, evidence from Bangladesh suggests that depressed mothers are over twice as likely to give birth to low-birth-weight infants, and their children are more likely to be underweight^(69)^ and over twice as likely to be stunted^(14)^. Peripartum depression has also been shown to decrease the odds of exclusive breast-feeding by 80 % in a Bangladeshi study^(70)^. Given these findings, it is likely that depression impacts many aspects of a woman’s health, and that of her family, as well as possibly being caused by poor food and nutrition security.

The burden of mental illness in LMIC has gained attention not only because of the magnitude of harm it causes, but also because of the lack of mental health services and treatment options currently available in these contexts^(18)^. While pharmacological treatment and psychiatric counselling may be out of reach or culturally unacceptable in some settings, community-based peer-support strategies are promising as both treatment and prevention options; however, they are still underdeveloped^(17^^,^^71^^)^. Such community-based, non-specialist programmes could go hand-in-hand with holistic health and nutrition interventions, such as homestead food production, known to be beneficial for a range of outcomes, including income generation, dietary improvements and increasing women’s agency^(18^^,^^71^^,^^72^^)^.

Most importantly, the high burden and clear detriments of both depression and poor indicators of food and nutrition security in settings such as Bangladesh require urgent action from the global health community and policy makers. Programmes and services that are embedded in local communities and integrated across various aspects of well-being may work to alleviate interlinked health burdens concomitantly.

## Conclusion

To date, the present study is the first to quantify the relationship between women’s mental health and indicators of food and nutrition security in South Asia, and to our knowledge in any LMIC setting. The high prevalence of depression in this area, linked with poor food access and diet quality as well as other factors such as lack of social support and poverty, is an important piece of evidence on which further studies and programmes in this field can build.

## Footnotes

* With limited birth records, and official birth records frequently incorrect, age can only be estimated approximately in adults. As such, some women who were initially reported to be less than 30 years of age were found to be older once detailed age estimation processes were used. Age alone after initial report was not an exclusion criteria.

## Supplementary material

For supplementary material accompanying this paper visit https://doi.org/10.1017/S1368980019003495. click here to view supplementary material
