# Supplementary material for: Depression among women of reproductive age in rural Bangladesh is linked to food security, diets and nutrition
Source: Public Health Nutr. 2020 Jan 9;23(4):660–73. doi: 10.1017/S1368980019003495 (PMC7058425; doi:10.1017/S1368980019003495)
Supplement: Supplementary file 1 [file S1368980019003495sup001.docx]

**APPENDIX A: Details on the indicators of food and nutrition security and potential confounders**

*Household food insecurity*

Household food insecurity was measured using the standard Household Food Insecurity Access Scale (HFIAS) from the Food and Nutrition Technical Assistance Project (FANTA) ^(^[^41^](#_ENREF_41)^)^. The HFIAS asks respondents whether an food insecurity related event happened and at what frequency in the last four weeks, and covers feelings of uncertainty or anxiety over food, perceptions of insufficient quantity and quality of food, reported reductions in food intake and consequences resulting from reductions ^(^[^41^](#_ENREF_41)^)^. The nine questions in the scale are ranked from least severe (worry about obtaining food) to most severe (going day and night without eating), and the frequency and severity of the event is used to categorize households as being food secure, or mildly, moderately or severely food insecure. In our study population, less than 10% of households were classified as being mildly food insecure, thus mild and moderate food insecurity groups were collapsed into one group, yielding three categories of household food insecurity instead of four.

*Household food consumption score (FCS)*

We also used the World Food Program’s food consumption score (FCS), adapted for the Bangladesh dietary pattern^(^[^64^](#_ENREF_64)^)^, as another measure of household food access. The FCS is a frequency-weighted and nutrient-weighted food frequency score based on an internationally standardized set of eight food groups, for which it is asked whether these were consumed by the household in the last seven days. This continuous score is categorized into four groups indicating poor, borderline, and acceptable low and acceptable high consumption, according to guidelines for Bangladesh ^(^[^64^](#_ENREF_64)^)^. In our analysis, the majority (86%) of the population fell into the acceptable categories, and thus we collapsed borderline and poor household consumption into one group, yielding a binary variable of poor/borderline compared to acceptable household diets.

*Women’s Dietary Diversity (WDDS)*

Dietary diversity information was collected through a 24-hour free-recall approach, followed by prompts for food groups not mentioned. The food items mentioned were coded into 21 pre-defined food categories by the enumerator, as well as noting whether the amount consumed in the last 24 hours was greater or less than 15 g. The 21 food categories were an expanded version of those included in FSNSP^(^[^65^](#_ENREF_65)^)^, as well as the validation study of the women’s dietary diversity score (WDDS) for use in Bangladesh ^(^[^44^](#_ENREF_44)^)^, together with the food groups included in the Minimum Dietary Diversity for Women of Reproductive Age (MDD-W) scale ^(^[^45^](#_ENREF_45)^)^. The WDDS quantifies the number of food groups eaten by an individual in the previous 24 hours, and thus is a proxy measure for both quantity (calories) and quality (micronutrients) of the food consumed. Although the WDDS is not validated for individual dietary assessment, it is used to determine population-based probability of meeting micronutrient adequacy in the diet^(^[^43^](#_ENREF_43)^)^.

In our analysis, we used both the linear score and the dichotomous indicators, and present associations at the population level. The WDDS can be consolidated into 13 groups with a minimum intake at or above 15 g (the 13R scale)^(^[^66^](#_ENREF_66)^)^. The 13R group scale was validated in Bangladesh by FANTA prior to the 10R group scale being completed. FANTA has proposed that eating five or more of 13 specified food groups most likely corresponds to adequate ingestion of all important micronutrients in Bangladesh ^(^[^45^](#_ENREF_45)^,^ [^67^](#_ENREF_67)^)^. Eating less than five food groups on the previous day indicates that the woman probably has a diet inadequate in micronutrients. We conducted all analyses using both the 13R and the 10R scale for comparison; both produced similar results. As the 13R group scale is more granular, we chose to use it to determine minimum dietary diversity.

We also examined several key food groups from the WDDS that provide essential micronutrients to analyze individually in addition to the full score indices. Since the average dietary diversity score was very low in this population, some food groups were not analyzed. Food groups that were eaten by at least 5% of the population on the previous day and were of particular interest for this analysis for their nutrient contributions were: dairy, eggs, fish, flesh/meat, dark green leafy vegetable, vitamin A-rich foods and vitamin C-rich foods. Vitamin C-rich and vitamin A-rich groups were originally four separate groups disaggregated by vegetables and fruits, but were collapsed for this analysis.

*Women's agency index construction*

The interview questions on women's agency fell in four domains: social support (support), ability to make decisions in the household (decisions), ability to leave the homestead (mobility), and community engagement and inter-personal communication (communication). For creating the indices, we used questions that could be answered by a majority of women and to which at least some women answered positively (>5%), and excluded questions only applicable to women with children. In each domain women could achieve a maximum of three points. When domains were comprised of many questions, each question was assigned points based on the answer and then simplified into three categories based on the distribution of responses. The scoring system for the agency index is described in Appendix B, Table 2.

The social support scale combined information on the frequency that a woman reported visiting her natal family, and on whether a woman reported the ability to seek help for various issues: ability to borrow money, borrow food if needed, get help when ill, and speak to someone about a problem.

For the decision-making domain, all women were asked about their ability to decide on issues: food preparation, large and small expenditures, visiting her natal family, health matters, and, if she earned any income, whether she could decide on how to use it. Very few women earned any income, but since having income is most likely indicative of some amount of agency, this aspect was retained as part of the score, and not earning any income was assigned ‘0’. Many women took no part in any listed decisions, and they comprised the lowest group.

The mobility domain was comprised of three questions: whether the woman left the homestead in the last month, whether she had visited a clinic by herself or with her children, and whether she, in theory, was able to leave the homestead by herself or only accompanied by children.

The communication score was determined by a series of questions on how comfortable a woman felt speaking out, how often she spoke to her husband, topics she discussed with her husband and how comfortable she felt speaking to other women about various topics. Most women were uncomfortable speaking to other women, so they were assigned one point if comfortable speaking about one or more of the issues listed, and zero if uncomfortable speaking about issues.

**APPENDIX B: TABLES**

**APPENDIX B, TABLE 1: Variable summary**

| **Definition of variables used in analysis** | |
| --- | --- |
| Women of reproductive age | 2,624 women were enrolled, 2,599 women completed the household and women’s modules during the baseline survey |
| Pregnant women | Self-reported pregnant in women’s questionnaire and/or reported to be pregnant during anthropometry and blood module |
| Postpartum women | Women who gave birth within the last 12 months |
| Peripartum women | Women who are pregnant and those who had a live birth or stillbirth in the last 12 months |
| Non-peripartum women | Women who are not self-reportedly pregnant or within one year postpartum |
| Major Depression | Screened positive for major depression (score of 12 or more on Edinburgh Postpartum Depression Scale [EPDS]) |
| Minor or major Depression | Screened positive for minor/major depression (score of 10 or more on EPDS) |
| **EXPOSURE TERMS** | |
| Household food insecurity | Self-reported food insecurity, measured by the Household Food Insecurity Access Scale (HFIAS), collapsed into 3 categories |
| Household Food Consumption Score | Food consumption score (FCS) from the Household Food Security Nutrition Assessment for Bangladesh^(^[^42^](#_ENREF_42)^)^, based on 9 frequency-weighted and nutrient-weighted food groups. Dichotomized into 2 categories: borderline/low vs. normal |
| Inadequate diet of women of reproductive age | Women consumed less than 5 food groups of the 13-food group Women’s Dietary Diversity Scale (WDDS) in previous 24 hours, excluding foods of which less than 15 g were consumed |
| Dietary diversity food groups | Women consumed more than 15 g of each food group selected for high micronutrient content in previous 24 hours (WDDS 13 groups, 15 g cut-off): Dairy, eggs, fish, flesh foods, dark green leafy vegetables, vitamin A-rich fruit and vegetables, vitamin C-rich fruits and vegetables |
| Anemia | Hemoglobin (Hb) <12.0 g/dl in non-pregnant women and Hb <11.0 g/dl in pregnant women, measured in capillary blood using the *Hemocue* 201 device |
| Chronic Energy Deficiency (CED) | Woman’s Body Mass Index (BMI) <18.5 |
| Severely or moderately thin | BMI <16 |
| Moderately thin | BMI 16 - 16.99 |
| Mildly thin | BMI 17 - 18.49 |
| Normal weight | BMI 18.5 - 24.99 |
| Overweight and obese | BMI ≥25 |
| **POTENTIAL CONFOUNDERS** | |
| Religion | Hindu or Muslim |
| Wealth | Household asset score determined by principle components analysis, weighted by family size using standard Demographics Health Survey (DHS) and FSNSP techniques^(^[^65^](#_ENREF_65)^,^ [^68^](#_ENREF_68)^)^, in quintiles |
| Household size | Total number of people regularly living and eating meals in the home, grouped into 1-4 members, 5-9 members or 10 or more members |
| Live births | Total number of live births based on woman's birth history |
| Age | Self-reported age (in years) of woman |
| Age at first marriage | Self-reported age (in years) of first marriage of woman |
| Education | Self-reported years of schooling completed: none, partial primary schooling, completed primary schooling, partial secondary education, and completed secondary education or higher |
| Women's literacy | Woman able to demonstrably read a simple sentence; classified as literate, partially literate and illiterate |
| Currently breastfeeding | Woman's self-report on current breastfeeding * |
| Duration of breastfeeding | Months since most recent birth in the last three years was included, with a dummy variable for women who did not give birth in the last three years, for whom the mean duration from the others was assigned |
| Support | Score of 1-3 calculated from frequency of contact with natal family and ability to ask others for help with various issues |
| Decisions | Score of 0-3 calculated from woman's ability to decide on household aspects such as expenditures, health and food preparation |
| Mobility | Score of 0-3 calculated from whether woman left homestead in the previous month, could leave if needed, at any time visited a health center alone or with children |
| Communication | Score of 1-3 calculated from frequency of speaking to husband, comfort speaking out publicly and comfort speaking to other women about issues such as health or education |
| Women's Agency Index | Adapted measures from DHS and used previously by Helen Keller International, or adapted from the Women’s Empowerment in Agriculture Index (WEAI)^(^[^68^](#_ENREF_68)^,^ [^69^](#_ENREF_69)^)^. Scale constructed of 4 agency dimensions: social support, decision-making, mobility, and inter-personal communication |

**APPENDIX B, TABLE 2: WOMEN'S AGENCY DOMAINS**

| **Social Support** | |
| --- | --- |
| Visiting natal family | 1 point for often vs. less often or never |
| Seeking help from someone:   - Lending money - Food assistance - If sick - To discuss personal problems | 1 point for each positive answer |
| **Decision-making** | |
| Ability to decide on issues:   - Food preparation - Large and small expenditures - Visiting her natal family - Health matters - If woman earned any income, whether she could decide on how to use it | 1 point for each positive answer |
| **Mobility** | |
| Leaving the homestead in the last month | 1 point for positive answer |
| Ever visiting a clinic by herself or with her children | 1 point for positive answer |
| In theory whether woman was able to leave the homestead by herself or only accompanied by children | 1 point for positive answer |
| **Communication** | |
| Comfort level speaking out on issues | 1 point for comfortable |
| Frequency woman spoke to her husband | 1 point for often or sometimes |
| Topics regularly discussed with husband:   - Work/agricultural activity - Events at home - Expenditures - Community events - Her own health - Child feeding and health | 1 point for each topic able to be discussed with husband |
| Comfort speaking to other women on various topics:   - Problems of the community - Education problems - Health problems - Problems of women - Information on health or nutrition | 1 point for one or more positive response |

| **ASSIGNMENT OF POINTS FOR WOMEN’S AGENCY DOMAINS** | | | |
| --- | --- | --- | --- |
| **SUPPORT** | **Amount of social support and contact with natal family** | | |
| **Overall support score:** | *Frequency of contact with natal family and ability to ask others for help with various issues* | | |
|  | 1. Little (0-3 points) | 433 | 17% |
|  | 1. Some (4 points) | 615 | 24% |
|  | 1. A lot (5 points) | 1551 | 60% |
| **DECISIONS** | **Ability of woman to decide on household and health matters** | | |
| **Overall decision score** | *Woman's ability to decide on HH aspects such as expenditures, health and food preparation* | | |
|  | 1. Unable (0 points) | 661 | 25% |
|  | 1. A little able (1 point) | 646 | 25% |
|  | 1. Somewhat able (2 points) | 566 | 22% |
|  | 1. Able (3-6 points) | 726 | 28% |
| **MOBILITY** | **Ability of woman to leave homestead** |  |  |
| **Overall mobility score** | *Physically left homestead in the previous month, could leave if needed, at any time visited a health center alone or with children* | | |
|  | 1. Unable (0 points) | 572 | 22% |
|  | 1. May be able (1 point) | 1027 | 40% |
|  | 1. Somewhat able (2 points) | 548 | 21% |
|  | 1. Able (3 points) | 452 | 17% |
| **COMMUNICATION** | **Ability of woman to discuss freely with husband and other women** | |  |
| **Overall communication score** | *Frequency of speaking to husband, comfort speaking out publicly and comfort speaking to other women about various issues such as health and education* | | |
|  | 1. Unable or very little (0-4 points) | 631 | 24% |
|  | 1. Somewhat able (5 points) | 1214 | 46% |
|  | 1. Able (6-8 points) | 754 | 29% |
| **Women's Agency Index** | **Categorical women’s agency score (combination of four domains above)** | | |
|  | *Very low* | 598 | 23% |
|  | *Low* | 421 | 16% |
|  | *Medium* | 515 | 20% |
|  | *High* | 456 | 18% |
|  | *Very high* | 609 | 23% |

**APPENDIX B, TABLE 3:**

| **Association of depression with indicators of food and nutrition security** | | | | | | | |
| --- | --- | --- | --- | --- | --- | --- | --- |
| NON-PERIPARTUM WOMEN | | | |  | PERIPARTUM WOMEN | | |
|  | BASE MODEL | MODEL 1 | MODEL 2 |  | BASE MODEL | MODEL 1 | MODEL 2 |
| **EXPOSURE** | **Crude** | **Adjusted** | **Adjusted** |  | **Crude** | **Adjusted** | **Adjusted** |
| **HH food insecurity** | n=1559 | n=1559 | n=1548 |  | n=1040 | n=1037 | n=1037 |
| **OR (95% CI)** | 2.54 (2.10-3.07) | 2.74 (2.16-3.47) | 2.50 (1.96-3.18) |  | 2.83 (2.19-3.67) | 3.22 (2.27-4.57) | 3.02 (2.12-4.30) |
| **P-value** | <0.001 | <0.001 | <0.001 |  | <0.001 | <0.001 | <0.001 |
|  |  |  |  |  |  |  |  |
| **HH food consumption** | n=1559 | n=1559 | n=1548 |  | n=1040 | n=1037 | n=1037 |
| **OR (95% CI)** | 3.37 (2.46-4.63) | 2.38 (1.64-3.45) | 1.72 (1.17-2.53) |  | 2.92 (1.94-4.39) | 2.44 (1.46-4.07) | 1.83 (1.09-3.05) |
| **P-value** | <0.001 | <0.001 | 0.002 |  | <0.001 | 0.001 | 0.021 |
|  | | | | | | | |
| **Minimum DD of Women** | n=1557 | n=1557 | n=1548 |  | n=1040 | n=1037 | n=1037 |
| **OR (95% CI)** | 1.96 (1.49-2.57) | 1.80 (1.31-2.46) | 1.49 (1.08 - 2.09) |  | 2.25 (1.54-3.28) | 1.99 (1.28-3.11) | 1.74 (1.10-2.76) |
| **P-value** | <0.001 | <0.001 | 0.016 |  | <0.001 | 0.002 | 0.018 |
|  | | | | | | | |
| **Anemia** | n=1505 | n=1505 | n=1503 |  | n=999 | n=999 | n=961 |
| **OR (95% CI)** | 0.97 (0.74-1.29) | 0.99 (0.73-1.35) | 1.02 (0.74-1.41) |  | 1.18 (0.85-1.66) | 1.19 (0.81-1.77) | 1.22 (0.82-1.84) |
| **P-value** | 0.855 | 0.946 | 0.915 |  | 0.326 | 0.372 | 0.324 |
|  | | | | | | | |
| **CED (BMI<18.5)** | n=1550 | n=1550 | n=1548 |  | n=550* | n=550* | n=550* |
| **OR (95% CI)** | 1.34 (1.04–1.73) | 1.40 (1.05-1.86) | 1.35 (1.01-1.82) |  | 1.11 (0.71-1.74) | 0.87 (0.51-1.51) | 0.83 (0.47-1.48) |
| **P-value** | 0.024 | 0.022 | 0.054 |  | 0.638 | 0.911 | 0.530 |
| ABRREVIATIONS: HH, household; OR, Odds Ratio; CI, Confidence Interval; DD, Dietary Diversity; CED, Chronic Energy Deficiency; BMI, Body Mass Index  *Only includes women between 2 months and 1 year postpartum, as per DHS guidelines on BMI for women. | | | | | | | |
| BASE MODEL: multilevel model with random intercepts at the cluster level | | | | | | | |
| MODEL 1: as BASE MODEL, also adjusting for age, age at first marriage, time since first marriage, religion, wealth, HH size, live births, stillbirth in last year, woman's education, literacy, breastfeeding status, duration of breastfeeding, whether the woman had never had a child before, woman's agency in four domains (mobility, support, decision-making and inter-personal communication), interviewer | | | | | | | |
| MODEL 2: as MODEL 1, also adjusting for other exposures (food insecurity, household food consumption score, minimum woman's dietary diversity, and woman's BMI) | | | | | | | |
|  | | | | | | | |

**APPENDIX B, TABLE 4:**

| **Relationship of depression with food groups eaten (versus not) on previous day** | | | | | |
| --- | --- | --- | --- | --- | --- |
| NON-PERIPARTUM WOMEN | | |  | PERIPARTUM WOMEN | |
|  | BASE MODEL | MODEL 1 |  | BASE MODEL | MODEL 1 |
| **EXPOSURE** | **Crude** | **Adjusted** |  | **Crude** | **Adjusted** |
| **Sample size** | n=1557 | n=1557 |  | n=1040 | n=1037 |
| **Dairy** | | | | | |
| **OR (95% CI)** | 0.73 (0.54-0.97) | 0.83 (0.59-1.15) |  | 0.47 (0.32-0.70) | 0.48 (0.30-0.75) |
| **P-value** | 0.032 | 0.255 |  | <0.001 | 0.002 |
| **Eggs** | | | | | |
| **OR (95% CI)** | 0.51 (0.33-0.78) | 0.57 (0.36-0.90) |  | 0.47 (0.26-0.86) | 0.56 (0.29-1.09) |
| **P-value** | 0.002 | 0.016 |  | 0.015 | 0.086 |
| **Fish** | | | | | |
| **OR (95% CI)** | 0.56 (0.42-0.74) | 0.58 (0.42-0.79) |  | 0.62 (0.42-0.92) | 0.60 (0.38-0.96) |
| **P-value** | <0.001 | 0.001 |  | 0.018 | 0.031 |
| **Meat** | | | | | |
| **OR (95% CI)** | 1.01 (0.65-1.56) | 1.16 (0.72-1.87) |  | 0.95 (0.54-1.66) | 1.27 (0.66-2.45) |
| **P-value** | 0.965 | 0.552 |  | 0.854 | 0.471 |
| **Dark Green Leafy Vegetables** | | | | | |
| **OR (95% CI)** | 1.14 (0.87-1.49) | 0.99 (0.73-1.35) |  | 0.77 (0.52-1.15) | 0.77 (0.50-1.21) |
| **P-value** | 0.354 | 0.993 |  | 0.203 | 0.260 |
| **Vitamin A-rich fruits and vegetables** | | | | | |
| **OR (95% CI)** | 0.60 (0.43-0.85) | 0.57 (0.39-0.84) |  | 0.93 (0.61-1.44) | 1.09 (0.66-1.80) |
| **P-value** | 0.004 | 0.008 |  | 0.755 | 0.725 |
| **Vitamin C-rich fruits and vegetables** | | | | | |
| **OR (95% CI)** | 0.64 (0.50-0.82) | 0.71 (0.54-0.94) |  | 0.61 (0.44-0.85) | 0.61 (0.41-0.89) |
| **P-value** | <0.001 | 0.018 |  | 0.003 | 0.011 |
| *ABBREVIATIONS: OR, Odds Ratio; CI, Confidence Interval* | | | | | |
| BASE MODEL: multilevel model with random intercepts at the cluster level | | | | | |
| MODEL 1: as BASE MODEL, also adjusting for age, age at first marriage, time since first marriage, religion, wealth, HH size, live births, stillbirth in past year, woman's education, literacy, breastfeeding status, duration of breastfeeding, whether the woman had never had a child before, woman's agency in four domains (mobility, support, decision-making and inter-personal communication), interviewer | | | | | |
